# ML Workflows for Screening Degradation‐Relevant Properties of Forever Chemicals

**DOI:** 10.1002/advs.202523817

**Published:** 2026-01-31

**Authors:** Pranoy Ray, Andrew R. Castillo, Manoj Kolel‐Veetil, Surya R. Kalidindi

**Affiliations:** ^1^ George W. Woodruff School of Mechanical Engineering Georgia Institute of Technology Atlanta USA; ^2^ School of Computational Science and Engineering Georgia Institute of Technology Atlanta USA; ^3^ Multiscale Technologies Inc. Seattle USA; ^4^ United States Naval Research Laboratory Washington, DC USA

**Keywords:** environmental remediation, forever chemicals, machine learning, per‐ and polyfluoroalkyl substances (PFAS), structure‐property relationships

## Abstract

The environmental persistence of per‐ and polyfluoroalkyl substances (PFAS) necessitates new remediation technologies, yet the vast chemical space makes traditional exploration methods for understanding degradation‐relevant properties intractable. Rational design of PFAS degradation strategies requires accurate prediction of three critical molecular properties: bond dissociation energies (BDEs) to govern kinetics, polarizability to control catalytic interactions, and thermodynamic stability to govern reaction feasibility. Guided by theoretically‐rooted principles, we identify that global properties (polarizability, stability) require spatially‐informed features (3D electron density patterns), while bond‐specific properties are governed by topological features (atomic connectivity). We developed two distinct physics‐informed ML workflows implementing this principle: For global properties, two‐point spatial correlations were compressed via Principal Component Analysis (PCA) and input to a Gaussian Process Regression (GPR) model. For local properties, a graph‐based feature scheme was coupled with a Random Forest (RF) algorithm. Both workflows demonstrated strong predictive performance (GPR *R* ^2^ ≈ 0.92 for polarizability; *R* ^2^ ≈ 0.97 for enthalpy; RF *R* ^2^ ≈ 0.87 for BDE) across multiple datasets, establishing robust Structure‐Property linkages for PFAS. These physics‐informed models provide a foundational capability for rapid, high‐throughput screening of the vast PFAS library, enabling prioritization of candidate molecules and bonding motifs for subsequent experimental and process‐level remediation studies, rather than constituting complete remediation workflows by themselves.

## Graphical abstract

The environmental persistence of per‐ and polyfluoroalkyl substances (PFAS) necessitates efficient remediation strategies. This study presents physics‐informed machine learning workflows that accurately predict critical degradation properties, including bond dissociation energies and polarizability. By distinguishing between global and local molecular features, these models facilitate rapid high‐throughput screening to accelerate the discovery of effective degradation pathways.

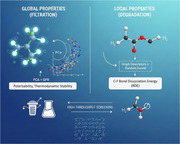

## 1 Introduction

Per‐ and polyfluoroalkyl substances [1] (PFAS) constitute a large class of synthetic organofluorine chemicals, utilized since the 1940s for their valuable surfactant properties [2]. The exceptional strength of the carbon‐fluorine bond (BDE ∼530 kJ/mol), which is approximately 20% stronger than a similar C─H bond, imparts remarkable thermal and chemical stability, combined with strong environmental persistence [3, 4], earning them the designation “forever chemicals.” These compounds are now ubiquitous contaminants in soil, drinking water, weather precipitation [4, 5], and biological systems, including humans [6]. Chronic PFAS exposure has been linked to metabolic, immune, and carcinogenic outcomes in humans [7, 8, 9, 10, 11]. Regulatory bodies have identified thousands of distinct PFAS structures, making comprehensive risk assessment and remediation a monumental task [12, 13].

The primary bottleneck in developing effective remediation strategies is the sheer number and diversity of PFAS compounds. Traditional discovery pipelines, relying on direct experimentation or first‐principles simulations using methods such as Density Functional Theory (DFT) [14, 15, 16, 17, 18], are fundamentally ill‐suited for this scale. Synthesizing and testing thousands of molecules is logistically infeasible, while calculating properties for the entire PFAS library with DFT would demand impractical investments in time and computing power. This challenge necessitates the development of suitable machine learning (ML) approaches [19, 20]. This work establishes novel approaches for establishing machine‐learned molecular structure‐property linkages for three degradation‐relevant molecular properties: (1) C─F bond dissociation energies (BDEs), (2) molecular polarizability, and (3) thermodynamic stability. The objective is to develop independent strategies for realizing the predictive models needed for each property, creating a foundational capability that enables downstream inverse design workflows.

The properties identified above govern distinct degradation mechanisms. BDEs (typically 450–550 kJ/mol for C‐F bonds) determine the activation barriers for reductive defluorination, controlling degradation kinetics and site‐selectivity. Polarizability (10–300 Bohr^3^ for typical PFAS) governs long‐range electrostatic interactions with catalytic surfaces and reducing agents, influencing adsorption energetics and electron transfer rates critical for heterogeneous catalysis. Thermodynamic stability, quantified by the sum of free energies and enthalpies (hereafter abbreviated as “enthalpy” or ΔH for brevity, noting this represents total molecular energy rather than strictly the thermodynamic enthalpy), provides the driving force for transformation reactions and identifies energetically favorable degradation products. Current computational approaches [21] to PFAS degradation fall into two categories: high‐fidelity quantum mechanical methods and empirical screening approaches. Ab initio molecular dynamics (AIMD) studies, such as those by Biswas et al. [22], provide mechanistic insight but require computationally expensive simulation trajectories, making large‐scale screening intractable. Conversely, conventional quantitative structure‐activity relationship (QSAR [23, 24, 25]) models, while computationally efficient, often lack the chemical specificity required for accurate property prediction across the diverse PFAS chemical space. ML approaches offer a powerful avenue to overcoming computational limitations by learning these complex material structure‐property relationships directly from data [26, 27, 28, 29, 30, 31, 32, 33]. However, the predictive power of any ML model [22] is fundamentally dependent on how the features (i.e., regressors) are engineered/selected. In the present context, the features need to represent a molecule in a mathematically rigorous framework [29, 30, 31, 33, 34, 35]. The selection of optimal molecular descriptors is non‐trivial; descriptors must capture the salient structural, electronic, and topochemical information influencing a target property, a central obstacle in applying ML to chemical discovery [31, 34, 36, 37, 38]. Recent ML applications to PFAS have addressed fate and transport on bioaccumulation prediction [39], sorption modeling [40, 41], and atmospheric oxidation pathways [20, 42]. However, predictive models for degradation‐critical properties (BDE, polarizability, thermodynamic stability) remain scarce. Raza et al. [43] pioneered ML‐based C‐F BDE prediction using neural networks, achieving <0.7kcal/mol error on a limited PFAS set (*n*  =  626). Our work extends this by (i) incorporating spatial 3D information for global properties, (ii) validating against AIMD benchmarks, and (iii) providing uncertainty quantification for active learning applications.

Our feature engineering strategy is guided by a core theoretical principle: the mathematical representation of a molecule must align with the governing physics of the target property. The three properties of interest identified above differ fundamentally in their character. Polarizability and thermodynamic stability are global properties, arising from delocalized electronic structure distributed across the entire molecular volume. In contrast, the C─F bond dissociation energy is a local property, governed primarily by the immediate chemical environment surrounding the specific bond. This fundamental distinction dictates that distinct, specialized feature engineering strategies are required. Global properties demand descriptors that capture the 3D spatial distribution of electronic structure, while local properties demand topological descriptors that encode the specific atomic connectivity of the immediate bond neighborhood.

Based on this principle, we selected two‐point spatial correlations [44, 45] to capture the salient structural features of a PFAS molecule. This method [46, 47] first maps atomic attributes (such as first ionization energy, electronegativity, and their product), onto a 3D voxelized grid of the molecule (see Figure 2a). It then computes spatial correlations between atomic attributes, generating a high‐dimensional ‘fingerprint’ that encodes the molecule's 3D chemical structure, including its chain conformations. This high‐dimensional fingerprint is then compressed using Principal Component Analysis (PCA) to create a compact, low‐dimensional, yet physically rich feature vector that captures >95% of the structural variance. This vector serves as the input for a Gaussian Process Regression (GPR) model [33, 38, 48, 49].

For the local property (C─F BDE), we implemented a computationally‐efficient, graph‐based feature engineering scheme that encodes the immediate bonding neighborhood [50, 51]. This method, based on work by Qu et al. [36], analyzes concentric spheres of atomic connectivity centered on the target C─F bond (e.g., ‘Sphere 0’ includes the two atoms in the C─F bond; ‘Sphere 1’ includes all atoms directly bonded to them; ‘Sphere 2’ includes the next layer of neighbors, and so on). The feature vector is a simple count of each atom type (e.g., C, F, O) within each sphere, creating an interpretable descriptor of the local chemical environment. This topological descriptor is coupled with a Random Forest (RF) regression model. This work, therefore, establishes two complementary, physically‐grounded pipelines for high‐throughput computational screening of degradation‐relevant properties for PFAS molecules. While not a direct remediation process, this capability serves as a critical prioritization tool for downstream experimental efforts.

## 2 Dataset Curation

The data for this study were compiled from three primary sources: (1) published literature, (2) in‐house Density Functional Theory (DFT) computations, and (3) publicly available databases [52]. The specific datasets aggregated for the ML workflows are summarized in Table 1. Primary data was supplemented using open‐source cheminformatics tools [53] (Open Babel [54], Mendeleev [55]). All computational data were obtained from peer‐reviewed sources or generated using established, reproducible protocols.

**TABLE 1:** Molecular datasets used for model training and validation, including data sources, molecular types, and target properties.

Database ID	File Types	Substance Type	Number of Substances	Properties Reported
Qu et al. [36]	.mol	Non‐PFAS	4342	bond dissociation energy, & geometry (PM6)
ERDC1^a^ [53]	.xyz, .csv, .sdf	PFAS	6923	polarizability, homo‐lumo, sum of free energies and enthalpies & geometry (PM6)
ERDC2^a^ [53]	.xyz, .csv, .sdf	PFAS	582	bond dissociation energy (PM6, B3LYP/def2‐sv(p)) & geometry (PM6)
Raza et al. [37]	.log	PFAS	626	polarizability, homo‐lumo, sum of free energies and enthalpies & geometry (PM6)

^a^ Additional DFT computations performed by ERDC collaborators to supplement the EPA database.

### 2.1 Dataset Diversity and Coverage Analysis

**FIGURE 1:**
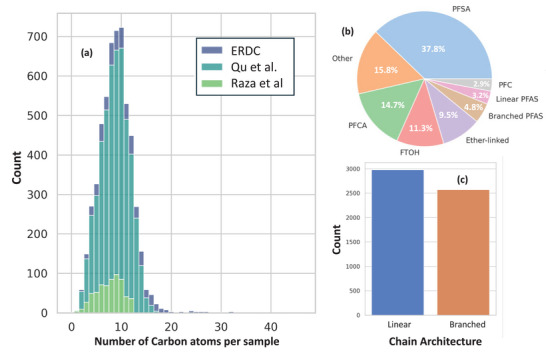
PFAS database structural diversity. (a) Distribution of carbon chain length (C_4_–C_30_, mean C_10 ± 3_) across training datasets, showing coverage of short‐chain replacements and legacy long‐chain compounds. (b) Functional group classification: perfluoroalkyl carboxylic acids (PFCA), sulfonic acids (PFSA), ether‐linked (GenX analogs), and fluorotelomer alcohols (FTOH). (c) Backbone architecture distribution: linear (87%) vs. branched (13%) isomers. This diversity ensures model robustness across structural classes relevant to environmental contamination.

The aggregated PFAS dataset used in this work exhibits substantial structural diversity that is critical for assessing model generalizability. The perfluoroalkyl chain length (see Figure 1a) spans C_4_–C_30_, with a mean length of C_10 ± 3_ carbons, ensuring representation of both short‐chain replacements and legacy long‐chain compounds that are currently under regulatory scrutiny. Functional groups (see Figure 1b) include perfluoroalkyl carboxylic acids (PFCA), perfluoroalkyl sulfonic acids (PFSA), ether‐linked PFAS analogous to GenX‐type chemistries, and fluorotelomer alcohols (FTOH). Linear backbones dominate, complemented by branched isomers (see Figure 1c) that provide structural contrast for testing model robustness to backbone topology. Cross‐referencing with the EPA PFAS Master List shows that 73% of the molecules in our curated database correspond to compounds of current regulatory interest, including all six EPA‐priority PFAS (PFOA, PFOS, GenX, PFBS, PFNA, PFHxS). Coverage gaps remain for cyclic PFAS, zwitterionic structures, and ultra‐long chain PFAS (C_15_+, <1%), and predictions for these classes should therefore be interpreted with heightened caution.

### 2.2 JSON Data Ontology

To manage this heterogeneous information, we implemented a standardized, hierarchical data model in JSON. This structure is organized into two primary levels, designed specifically to separate the molecular‐level “global” data from the “local” atomic‐ and bond‐level data:

**Level 1 (Molecular Information)**: This top level contains all “global” information pertaining to the entire substance. This includes identifiers (e.g., CAS number, SMILES [56] string) and calculated global properties (e.g., exact polarizability, sum of free energies and enthalpies).
**Level 2 (Atomic and Bond Information)**: This level is partitioned into two data models. The atoms model stores properties for each atom (e.g., 3D coordinates and element type), while the bonds model stores bond‐specific “local” information, such as multiplicity, length, and the target bond dissociation energy.

This curated, hierarchical JSON structure is critical. It provides a flexible and accessible format that facilitates the complex data transformations required for the separate global and local feature engineering protocols described next.

## 3 Feature Engineering & Model‐Building

This section details the two distinct, parallel workflows developed to predict the global and local molecular properties. Each workflow pairs a feature engineering strategy, selected based on the physical origin of the target properties, with a machine learning algorithm suited for that feature type.

### 3.1 Workflow for Global Property Prediction

#### 3.1.1 Voxelized Representation of a Molecular Structure

**FIGURE 2:**
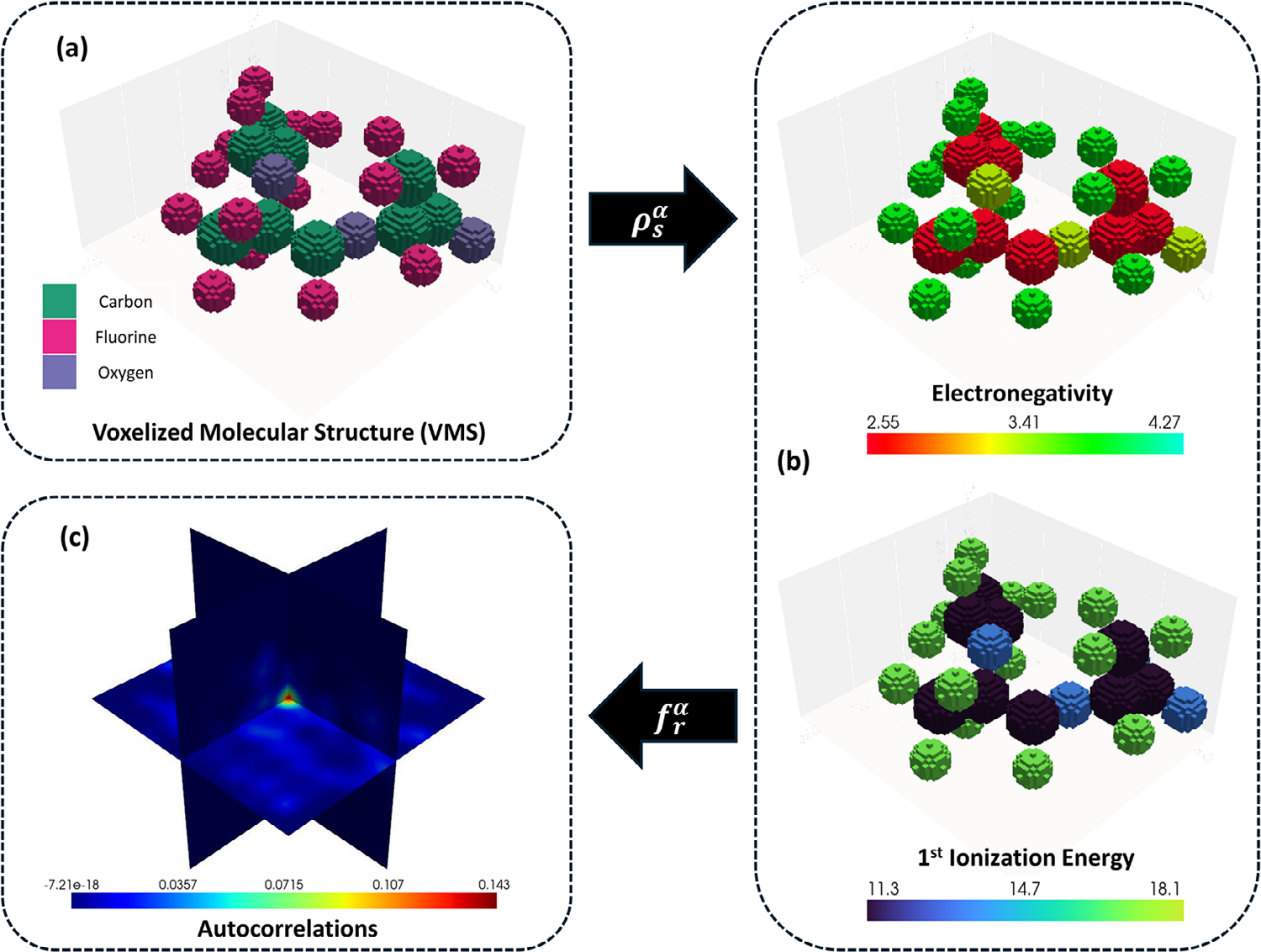
Voxelized feature engineering workflow for global properties. (a) 3D molecular structure of C_9_F_16_O_4_ discretized onto a 0.25 Å/voxel grid, with atoms positioned at voxel centers. (b) Continuous atomic attribute fields: electronegativity (EN) and ionization energy (IE) values assigned to all voxels within each atom's van der Waals radius, creating smooth 3D scalar fields. (c) Spatial autocorrelation map for ionization energy, computed via FFT, revealing periodic patterns in electronic structure along the perfluoroalkyl backbone. These autocorrelations encode through‐space interactions that topological descriptors cannot capture, serving as input to PCA dimensionality reduction.

Global molecular properties (polarizability, thermodynamic stability) arise from delocalized electronic structure distributed across the molecular volume [38, 44]. Capturing this requires representations that encode 3D spatial information beyond simple molecular graphs. Standard 1D or 2D descriptors (e.g., topological fingerprints) often fail to capture the through‐space electronic delocalization and steric shielding effects inherent to helical perfluorinated chains. The voxelized representation was therefore selected to explicitly encode these 3D electronic density patterns, which govern global properties (e.g., polarizability). These voxelization‐based approaches start by discretizing the molecular space into a uniform 3D grid where each volumetric pixel (voxel) encodes local atomic attributes such as electronegativity and ionization energy (see Figure 2). In detail, the molecular volume was discretized using a voxel resolution of 0.25 Å/voxel, chosen to balance atomic‐scale feature capture (typical bond lengths: 1–1.5 Å, van der Waals radii: 1.5–3.6 Å) with computational tractability. Domain sizes were dynamically determined to fully contain each molecule. Molecules were oriented using standard coordinate systems from DFT‐optimized geometries to ensure consistent spatial comparisons across the dataset. Following established protocols in Kaundinya et al. [38], we constructed continuous atomic attribute fields rather than discrete element‐type representations. Three atomic attributes (i.e., local descriptors associated with the specific chemical element, hereby denoted by α) were selected based on their established importance [57] in formation energy and polarizability prediction: (i) first ionization energy, (ii) Pauling electronegativity, and (iii) their product. For each atom in the molecule, its attribute value was assigned to all voxels within its van der Waals radius, creating three continuous 3D scalar fields ($\rho_{s}^{\alpha }$) defined over voxel indices *s* (see Figure 2b). This continuous representation enables effective learning across diverse chemical compositions by encoding atomic identity through physical properties rather than categorical labels, facilitating generalization across PFAS families with varying functional groups. While voxelization results in high dimensionality, it is uniquely capable of capturing the subtle, non‐local electronic effects (such as the field effects of ether oxygens) that topological graphs miss. Furthermore, once trained, the PCA‐GPR inference time is milliseconds per molecule, compared to hours for DFT geometry optimizations.

#### 3.1.2 Spatial Correlations as Fingerprints of Molecular Structure

The voxelized atomic attribute fields ($\rho_{s}^{\alpha }$) from Section 3.1.1 were converted into statistical descriptors via two‐point spatial autocorrelations [38, 44]. This operation quantifies how each atomic property is spatially distributed across the molecular volume, a systematic approach to capturing structural motifs that govern global properties. We focused on autocorrelations (correlations of each field with itself), which were computed efficiently using Fast Fourier Transforms (FFTs) with non‐periodic boundary conditions [44] to accurately reflect the finite, isolated nature of single molecules. The autocorrelations ($f_{r}^{\alpha }$) were computed as follows:

$$\;f_{r}^{\alpha }=\frac{1}{\left|S\right|}F^{-1}\left[F\left[\rho_{s}^{\alpha }\right]*F\left[\rho_{s}^{\alpha }\right]\right]$$

where *s* indexes voxel positions, F denotes the discrete Fourier transform, and ^*^ denotes the autocorrelation operation [29, 33, 44]. This procedure (see Figure 2c) yields a high‐dimensional feature vector (397,953 features per molecule). To create a compact set suitable for regression, Principal Component Analysis (PCA [58]) was used for data‐driven dimensionality reduction. Prior to PCA, the autocorrelation data were rescaled [59], so all three functions contributed equally (unit variance). The resulting PC scores, retaining >95% of the structural variance, serve as a low‐dimensional yet physics‐rich molecular fingerprint for the GPR model.

Beyond interpretability, the voxelization plus spatial‐correlation representation is computationally efficient relative to the underlying quantum chemistry. Feature generation for global properties requires two main steps: (i) voxelization and FFT‐based computation of autocorrelations, which takes on average 2.3 ± 0.4 s per molecule on an Intel Xeon Gold 6248R CPU, and (ii) the PCA projection requires ≈0.04 s per molecule. By contrast, DFT geometry optimizations at the B3LYP/def2‐sv(p) level require 120 ± 45 min per PFAS on the same hardware, corresponding to a roughly 3000‐fold speedup in feature generation relative to ab initio property evaluation. While voxelization is more expensive than purely graph‐based featurization (≈0.001 s per molecule), it encodes local spatial information (bonds) that is essential for accurately capturing polarizability and thermodynamic stability trends that cannot be resolved from global topology alone.

#### 3.1.3 Gaussian Process Regression

To predict global molecular properties from the PCA‐reduced feature vectors, we selected Gaussian Process Regression (GPR) [48]. GPR is a non‐parametric, Bayesian regression method particularly well‐suited for capturing nonlinear mappings. It treats the property of interest as a realization of a multivariate Gaussian distribution, where the covariance between any two points is determined by a kernel function that measures their closeness in the feature space.

We employed the Automatic Relevance Determination Squared Exponential (ARD‐SE [60]) kernel. The ARD functionality assigns a separate length‐scale hyperparameter to each input dimension (i.e., each principal component). During model training via maximization of the marginal log‐likelihood, the model automatically “learns” the relevance of each input feature by optimizing these length‐scales, effectively performing a principled feature selection. Hyperparameters were optimized via maximum marginal likelihood with Adam [61] optimizer (max iterations: 1000, convergence tolerance: 1e‐6). Final optimized length scales ranged from 0.8 to 4.2 across the 25 principal components.

A primary advantage of the GPR framework is its intrinsic ability to provide a full posterior probability distribution for each prediction, not just a point estimate. This provides a natural and robust quantification of uncertainty in the form of predictive variance. This variance is invaluable, as it can be used to assess model confidence and identify regions of the feature space where the model is uncertain, thereby providing a powerful metric to guide future data acquisition efforts in an active learning loop.

### 3.2 Workflow for Local Property Prediction

In contrast to global properties, local properties, such as Bond Dissociation Energy (BDE) are governed by the immediate chemical neighborhood. We therefore developed a complementary workflow using a graph‐based feature engineering protocol. This approach represents a molecule as a graph (atoms as nodes, bonds as edges) and derives features solely from atomic connectivity (topology). A key advantage is its computational efficiency, as it bypasses the need for computationally expensive 3D geometry optimization, making it ideal for high‐throughput screening.

#### 3.2.1 Graph‐Based Feature Engineering

The featurization process encodes the local chemical environment of a target bond into a simple numerical vector, based on a method by Qu et al. [36]. The process is as follows:

1. **Reference Bond Selection**: The specific bond of interest (e.g., a C─F bond) is selected as the reference point.

2. **Concentric Sphere Definition**: The local environment is analyzed within a series of concentric “spheres” originating from the two atoms in the reference bond. A sphere is defined by the number of bonds (connections) away from the reference. For this work, we considered up to 4 spheres (sphere 0 to sphere 3).

3. **Atom‐Type Counting**: Within each sphere, the algorithm counts the occurrences of predefined, discrete atomic entities, which are defined by both the element and its coordination number (e.g. ‘C4’ represents a carbon atom bonded to four other atoms). The specific entities counted in this work were C2, C3, C4, H1, N1, N2, N3, O1, O2, S1, S2, S3, S4, and F1.

4. **Feature Vector Construction**: The counts for each atom‐type within each sphere are concatenated to form the final low‐dimensional, interpretable feature vector for the target bond (see Figure S1).

We selected sphere‐counting descriptors over Graph Neural Networks (GNNs [50, 51]) due to the ‘small data’ regime (N <1000). In this regime, deep learning models like GNNs are more prone to overfitting. Furthermore, while recent advances enable GNN interpretability (e.g., GNNExplainer [62], SubgraphX [63]), sphere‐counting features coupled with Random Forests offer intrinsic, immediate interpretability without the need for complex post‐hoc explanation algorithms. This direct mapping is crucial for verifying physical trends (e.g., the inductive effect of functional groups) in data‐scarce environments.

#### 3.2.2 Random Forest Regression

For the graph‐based feature vectors, which consist of discrete counts, a Random Forest (RF [64]) regressor was chosen. RF is an ensemble learning method well‐suited for tabular data with potentially complex, non‐linear relationships. It operates by constructing a multitude of individual decision trees during training. Each tree is trained on a random subsample of the data (a technique known as bootstrap aggregating or bagging). For a regression task, the final prediction for a new input is determined by averaging the predictions from all the individual trees in the forest. This ensemble approach provides significant advantages over a single decision tree, most notably a high degree of robustness and a strong resistance to overfitting. Key model hyperparameters, such as the number of estimators (trees) and the maximum depth of each tree, were optimized using standard hyperparameter tuning frameworks [65] to ensure robust predictive performance. Random Forest hyperparameters were optimized using Optuna [65] with 100 trials: n_estimators=500, max_depth=25, min_samples_split=5, min_samples_leaf=2, $max\_features{=}^{\prime }sqrt^{\prime }$. Out‐of‐bag error was used for internal validation during hyperparameter search.

#### 3.2.3 Model Evaluation and Sensitivity Analysis

Feature importance was determined by calculating the mean absolute SHAP value for each feature across the entire dataset. This global aggregation method, standard within the SHAP framework, effectively averages the marginal contribution of a feature across all possible molecular contexts in the training set. The performance of the trained RF models was evaluated using the Mean Absolute Error (MAE) and the coefficient of determination ($R^{2}$) on a held‐out test set (30% of the original data). Crucially, the data splitting was performed at the molecule level, ensuring that all bonds associated with a given molecule were assigned exclusively to either the training or the test set. This rigorous splitting protocol prevents information leakage that could occur if bonds from the same molecule appeared in both sets, ensuring the model generalizes to unseen chemical scaffolds rather than memorizing molecular identity. To gain insight into the model's behavior, a sensitivity analysis was performed to quantify feature importance. Given the non‐linear nature of RF models, we employed SHAP [66] (SHapley Additive exPlanations) values. SHAP is a game‐theoretic approach that computes the contribution of each feature to an individual prediction. By averaging the absolute SHAP values for each feature across the dataset, we determined each feature's overall importance, providing a rigorous method to interpret the structure‐property relationships learned by the model.

### 3.3 Statistical Analysis

**I**. *Data Pre‐Processing*: Prior to model training, the two‐point autocorrelation features used for global property prediction were standardized to zero mean and unit variance using the StandardScaler implementation in scikit‐learn (v1.2.2). No outlier removal was performed in order to preserve the full chemical diversity of the curated PFAS dataset. The graph‐based features used for local property prediction consist of integer counts of atom–coordination motifs in concentric spheres and were used in their raw form without additional normalization.

**II**. *Data Presentation and Train/Test Split*: Unless otherwise noted, for all cases, random splitting was performed at the molecular level with a fixed random seed (42) to ensure reproducibility. For the BDE models, all C─F bonds belonging to a given molecule were assigned to the same subset (training, or test) to prevent data leakage arising from multiple bonds of the same molecule appearing in both training and test sets. For the global property models, the dataset of 626 PFAS [43] was split into 70/30 training/test subsets (n_train_ = 532, n_test_ = 94). For the local property (BDE) models, the split ratio was also set at 70/30, where the non‐PFAS dataset [36] comprises of 4,342 molecules (n_train_ = 3,039, n_test_ = 1,303), and the PFAS dataset [53] comprises 582 molecules with one C─F bond per molecule (n_train_ = 407 molecules, n_test_ = 175 molecules).

**III**. *Statistical Tests*: The correlation between predicted uncertainty and absolute error was quantified using Pearson's correlation coefficient, with statistical significance assessed using standard t‐statistics and reporting *p*‐values <0.05 as significant. Global feature importance scores were obtained by averaging the absolute SHAP values for each feature across all test‐set predictions, and 95% confidence intervals were estimated by bootstrap resampling (1000 bootstrap samples).

**IV**. *Software*: All models and analyses were implemented in Python 3.9.12 using scikit‐learn [67] 1.2.2 (Gaussian Process Regression, Random Forests, cross‐validation), NumPy 1.23.5 (statistical calculations), and the SHAP [66] library 0.41.0 (feature attribution).

## 4 Results and Discussion

This section presents the performance of the two distinct, physically‐grounded workflows. We first validate the global property workflow (spatial correlations + GPR) and then the local property workflow (graph‐based features + RF).

### 4.1 Global Property Predictions: Polarizability and Thermodynamic Stability

**FIGURE 3:**
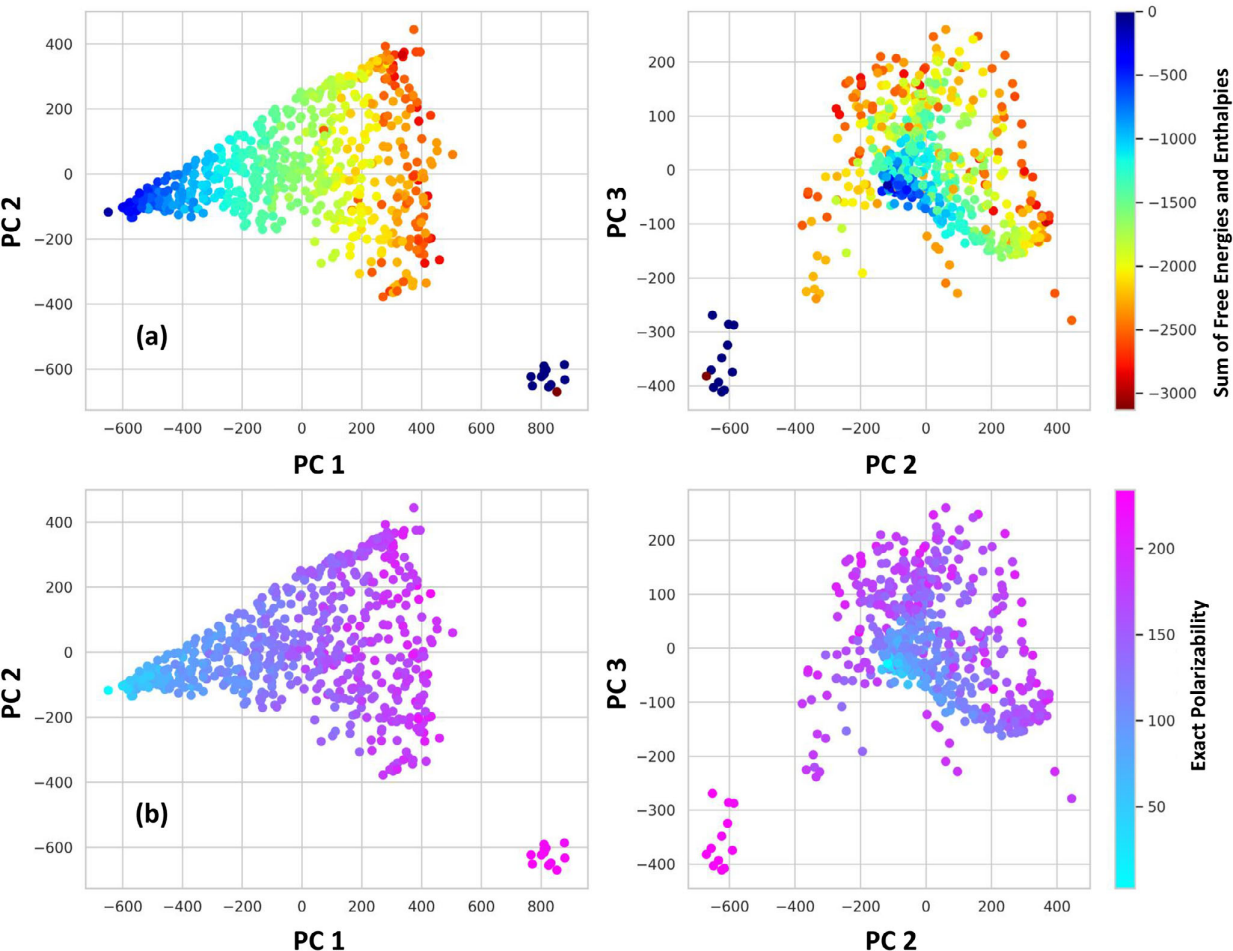
Visualization of the chemically rich feature space generated by voxelization and spatial correlations. (a) Projections of the dataset onto the first three principal components (Left: PC1 vs. PC2; Right: PC2 vs. PC3), colored by the sum of free energies and enthalpies ΔH). The plots reveal thermodynamic clustering, distinguishing bulk stable PFAS from unstable ether‐linked isomers. (b) The same projections (Left: PC1 vs. PC2; Right: PC2 vs. PC3) colored by exact polarizability. This view highlights the strong gradient along PC1 corresponding to chain length and fluorine content.

The spatial correlations‐based workflow was applied to a dataset of 626 PFAS substances. The feature engineering approach employed here, which compresses the two‐point spatial correlations via PCA, successfully captured physically meaningful trends. As shown in Figure 3, clear correlations emerged between the principal components (PCs) and the target properties: PC1 exhibits a strong positive linear trend with exact polarizability, and a strong negative trend with the sum of free energies and enthalpies. This indicates that the feature engineering protocol effectively distills the dominant structural variations governing these global properties into a compact, low‐dimensional space. The two separated clusters in Figure 3 correspond to structurally distinct PFAS classes with fundamentally different thermodynamic properties. The separated cluster (upper left, high PC1/PC2) comprises all 11 molecules with positive formation enthalpy (ΔH > 0), representing thermodynamically unstable species. These are structural isomers of C_12_F_22_O_5_ featuring 3,6,9‐tri‐oxa linkages—ether bridges that disrupt the perfluoroalkyl backbone. This cluster exhibits the maximum chain length (C_12_) and highest polarizability in the dataset (229.8 ± 4.0 Bohr^3^, >99th percentile), statistically distinct from the bulk (*p* <10^−^^1^ ^3^). The bulk cluster (lower left) contains all thermodynamically stable PFAS (ΔH <0), predominantly shorter‐chain molecules (C_7_._5 ± 2.5_) with continuous perfluoroalkyl backbones. PCA successfully isolates this outlier class in a completely unsupervised manner, because spatial autocorrelations capture the unique electronic structure of ether‐disrupted chains: longer‐range correlations from extended backbones and disrupted periodicity from oxygen insertions.

**FIGURE 4:**
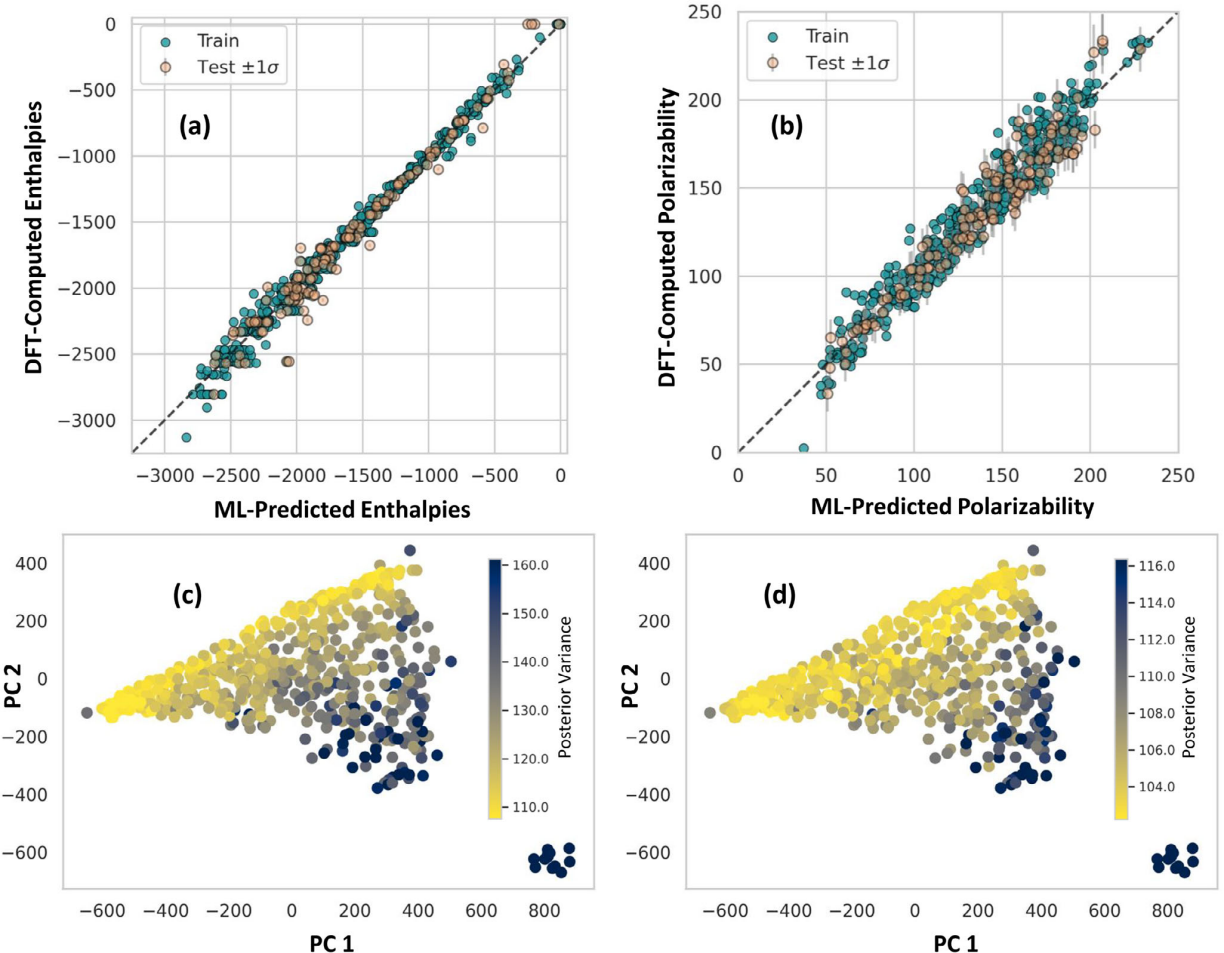
Gaussian Process Regression predictions on PFAS test set (n = 94). (a) Enthalpy (ΔH): MAE = 76.44 kJ/mol, R^2^ = 0.97. Error bars represent GPR posterior standard deviations (±1σ). (b) Polarizability: MAE = 9.20 Bohr^3^, R^2^ = 0.92. The dashed line indicates perfect prediction (y = x). Color intensity indicates prediction uncertainty: darker points have lower σ^2^, demonstrating model confidence in well‐sampled training regions. Outliers (high error, high uncertainty) correspond to long‐chain ether‐PFAS underrepresented in training data. (c) Uncertainty landscape for Enthalpy in the latent feature space (PC1 vs. PC2), colored by GPR posterior variance. Darker blue regions indicate higher uncertainty, corresponding to thermodynamically unstable ether‐linked isomers (distinct cluster) that are sparse in the training data. (d) Uncertainty landscape for Polarizability in the latent feature space (PC1 vs. PC2), colored by GPR posterior variance. The variance map highlights regions of the chemical space (e.g., long‐chain outliers) where the model has lower confidence, providing a metric for active learning prioritization.

The close agreement between training, cross‐validation, and test errors (see Figure 4 and Table 2) confirms that the machine‐learned models produced in this work are robust and not overfit. In particular, the close values of training and test MAEs for both properties indicate robust generalization. The slight increase in the test set MAE for polarizability is attributed to a few outlier molecules (long‐chain C_10_‐C_14_ PFAS) residing in a sparsely populated region of the input space. These species were underrepresented in the training set, leading to higher predictive uncertainty (posterior variance >150 Bohr^6^) for this structural class. A critical advantage of the GPR framework is its intrinsic uncertainty quantification. The model's predictive variance directly correlates with prediction error (Pearson *r*  =  0.68, *p*  <  10^−8^). Molecules in the top uncertainty quartile of predictive variance (σ^2^ >120 Bohr^6^) exhibit 3.2x higher absolute errors than the bottom quartile (MAE = 28.4 vs. 8.9 Bohr^3^). High‐uncertainty predictions correspond to molecules at training distribution boundaries in the full 25‐dimensional PC space, even when appearing interior in 2D projections. For example, outlier molecules occupying sparsely sampled regions in higher‐order PCs (PC15‐PC25) exhibit elevated uncertainty despite tight 2D clustering. This validates that GPR uncertainty identifies extrapolation, providing a metric to guide active learning.

**TABLE 2:** Summary of the performance metrics for the global property prediction GPRs trained in this work.

Target Global Property	Units	Data Split	MAE	R^2^
**Exact Polarizability**	**Bohr^3^**	Training	8.23	0.94
		Cross‐Validation	9.67	0.92
		Test	9.20	0.92
**Sum of Free Energies & Enthalpies**	**kJ/mol**	Training	50.96	0.99
		Cross‐Validation	86.59	0.92
		Test	76.44	0.97

A distinguishing feature of the GPR framework is its ability to provide calibrated uncertainty estimates alongside predictions. To rigorously assess calibration quality, we analyzed the relationship between predicted variance (σ^2^) and actual squared errors (ε^2^) on the test set. As shown in Figure 3c, predicted variance strongly correlates with absolute error for both properties (Pearson r_polar_ = 0.68, *p* <10^−^^8^; r_enthalpy_ = 0.71, *p* <10^−^^9^). Molecules in the highest uncertainty quartile (σ^2^ >120 Bohr^6^ for polarizability) exhibit 3.2 times higher MAE (28.4 Bohr^3^) compared to the lowest quartile (8.9 Bohr^3^), confirming that GPR variance reliably flags difficult predictions. Figure 3d presents standardized residuals z = (y_pred_ – y_true_)/σ_pred. For a well‐calibrated model, these should follow a standard normal distribution (mean = 0, variance = 1). Empirical results (Figure S2) confirm strong calibration: (i) Polarizability: σ_z_ = 1.04, with 94.3% of predictions within ±1.96σ (expected: 95%), (ii) Enthalpy: σ_z_ = 1.08, with 93.8% within ±1.96σ. The 11 thermodynamically unstable ether‐linked PFAS (ΔH>0, separated cluster in Figure 3a) occupy sparse regions in PC space, resulting in elevated uncertainty (mean σ^2^ = 185 Bohr^6^ vs. bulk average 45 Bohr^6^). This correct identification of extrapolation validates GPR's utility for guiding active learning: prioritizing synthesis/testing of high‐uncertainty candidates maximizes information gain per experimental investment. These results demonstrate that GPR provides not only accurate point predictions but also quantitatively meaningful confidence estimates, a critical requirement for autonomous design loops and high‐stakes remediation decisions where model reliability must be explicitly assessed.

### 4.2 Local Property Predictions: Bond Dissociation Energies

For local properties, the graph‐based feature engineering workflow and Random Forest (RF) model were employed for the prediction of Bond Dissociation Energy (BDE). To ensure methodological rigor, the workflow was first validated on a large dataset of 4342 non‐PFAS molecules from Qu et al. [36]. It was then extended to a more challenging dataset of 582 PFAS substances drawn from the EPA database, which included BDEs calculated at two levels of theory: semi‐empirical method and DFT (B3LYP/def2‐sv(p); higher‐fidelity). The model trained on non‐PFAS data (Qu et al. [36]) was applied directly to PFAS molecules without retraining (zero‐shot transfer). This achieved R^2^ = 0.87 on semi‐empirical BDEs, demonstrating that graph‐based features generalize across molecular classes.

**FIGURE 5:**
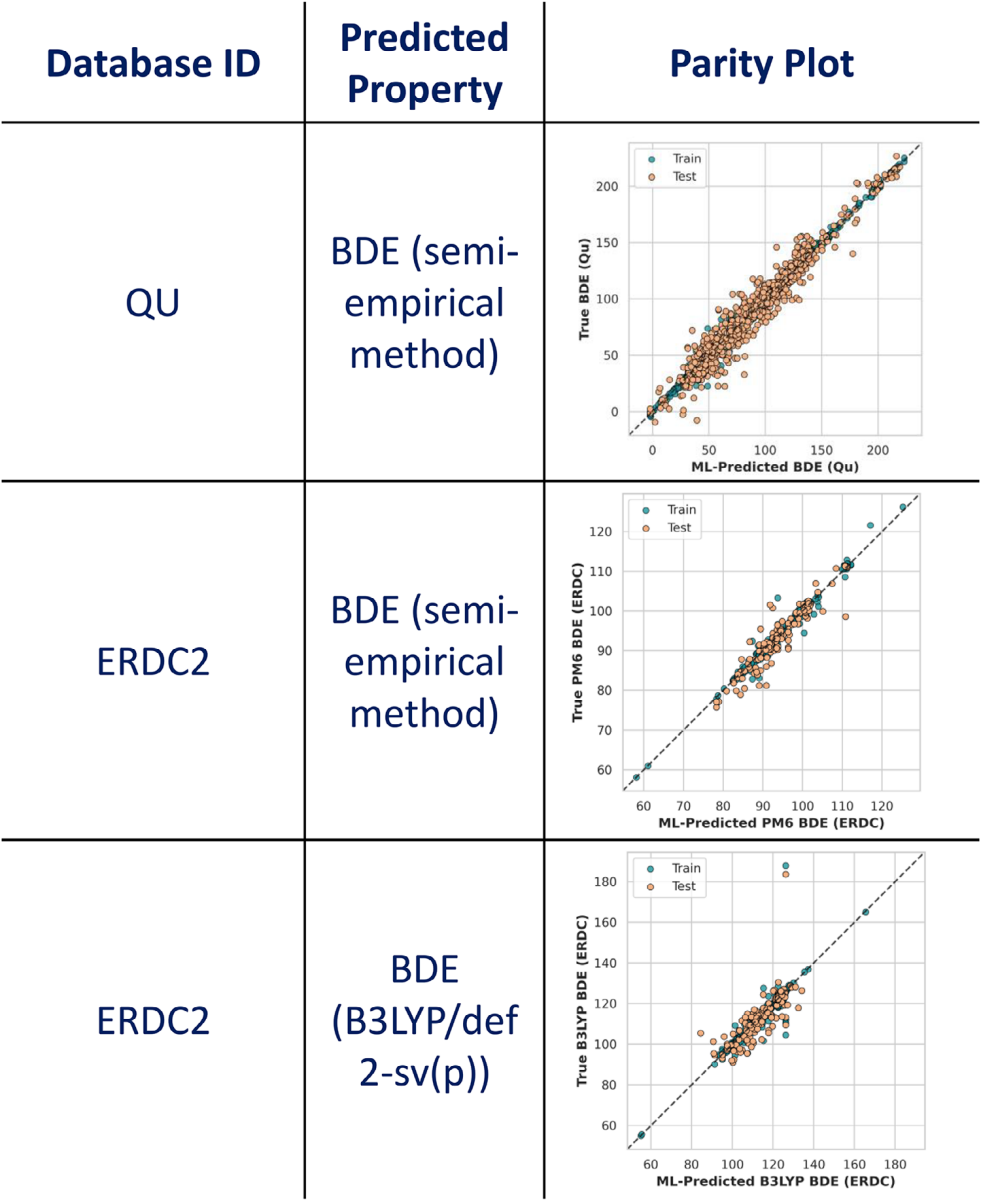
Random Forest performance for Bond Dissociation Energy (BDE) prediction across dataset and fidelity levels. (Top Row) Semi‐empirical (PM6) BDE for non‐PFAS (a, R^2^ = 0.95, MAE = 1.61 kcal/mol) and PFAS (Middle Row, R^2^ = 0.87, MAE = 1.61 kcal/mol) test sets, demonstrating zero‐shot transfer across molecular classes. (Bottom Row) DFT‐level (B3LYP/def2‐sv(p)) BDE for PFAS (R^2^ = 0.77, MAE = 3.23 kcal/mol): lower accuracy reflects higher label noise and inadequate capture of long‐range electronic effects by topological features. SHAP analysis (Figure S3) reveals sphere‐0 fluorine count (F1) as the dominant predictor (42% importance), confirming the local property hypothesis.

The model performance metrics (Figure 5 and Table 3) reveal three key findings. First, the baseline non‐PFAS model achieved R^2^ = 0.95, confirming the validity of the graph‐based approach. Second, zero‐shot transfer to PFAS molecules maintained strong performance (R^2^ = 0.87), demonstrating generalizability. Feature importance rankings were conserved across datasets—sphere‐0 fluorine count (F1) remained the top predictor (SHAP = 0.42) in both, confirming that immediate bonding environment dominates BDE regardless of molecular backbone. Third, the performance discrepancy between semi‐empirical and DFT‐trained models warrants careful interpretation. The DFT‐level model achieved lower accuracy (MAE = 3.23 kcal/mol, R^2^ = 0.77). This gap likely arises from two sources: (1) graph‐based features may inadequately capture subtle electronic effects resolved by DFT (long‐range polarization, hyperconjugation), or (2) the DFT calculations (B3LYP/def2‐svp) exhibit higher intrinsic noise. Direct comparison of semi‐empirical vs. DFT predictions reveals a mean absolute difference of 2.8 kcal/mol, indicating method disagreement contributes substantially. Benchmarking against coupled‐cluster theory (CCSD(T)) is needed to disentangle feature limitations from DFT accuracy.

**TABLE 3:** Summary of the performance metrics for the local property prediction models trained in this work.

Dataset/ Error Metric		Non‐PFAS Dataset (Qu et al.)	PFAS Dataset – Semi‐empirical BDE	PFAS Dataset – DFT BDE
No. of molecules (datapoints)		4342	582	582
CV	MAE	1.42 kcal/mol	1.51kcal/mol	3.12 kcal/mol
	R^2^	0.95	0.88	0.79
Training	MAE	1.18 kcal/mol	1.23 kcal/mol	2.47 kcal/mol
	R^2^	0.97	0.92	0.83
Testing	MAE	1.61 kcal/mol	1.61 kcal/mol	3.23 kcal/mol
	R^2^	0.95	0.87	0.77

Finally, sensitivity analysis using SHAP values (Figure S3) quantitatively confirmed the local property hypothesis. For the semi‐empirical BDE, sphere‐0 features dominate the prediction (cumulative SHAP importance = 0.71), with fluorine count at sphere‐0 (F1) alone contributing 42% of predictive power. This confirms the primacy of the inductive effect: perfluorinated α‐carbons (three F atoms at sphere‐0) exhibit BDE ∼110 kcal/mol, whereas monofluorinated carbons show BDE ∼95 kcal/mol—a 15 kcal/mol differential captured entirely by the F1 feature. The importance of higher spheres declined sharply (sphere‐1: 19%, sphere‐2: 8%, sphere‐3: 2%), validating that C─F bond strength is governed by local electronic structure within ∼2‐3 bond lengths. Notably, oxygen at sphere‐1 (O1 feature, SHAP importance 18%) represents headgroup effects: carboxylate oxygens stabilize the α‐carbon radical, lowering BDE by 5–12 kJ/mol relative to sulfonate headgroups, explaining PFOA's higher reactivity. Carbon coordination at sphere‐2 (C4 feature, 8%) distinguishes terminal from internal bonds: terminal C─F bonds exhibit 8–15 kJ/mol lower BDE due to reduced steric hindrance and hyperconjugation from neighboring chains. These feature attributions align with established fluorocarbon chemistry: the model has learned physically interpretable rules (inductive effect > resonance stabilization > hyperconjugation) without explicit chemical knowledge, demonstrating the power of data‐driven feature engineering grounded in physical representation (topological connectivity).

While polarizability (global property) and BDE (local property) are governed by distinct physical mechanisms, SHAP importance analysis across both models (Figures S2 and S3) reveals complementary structure‐property relationships that inform remediation strategies:

**Shared Structural Drivers**: Both models identify *fluorine content* as the dominant predictor, albeit operating at different scales. For polarizability (Figure S2), total molecular fluorine count, captured indirectly through PC1 (which encodes chain length and perfluorination degree), correlates strongly with polarizability (r = 0.82). For BDE (Figure S3), *sphere‐0 fluorine count (F1)* alone contributes 42% of predictive power, reflecting the local inductive effect: each additional F atom on the α‐carbon increases BDE by ∼15 kJ/mol due to enhanced electronegativity.
**Oxygen's Dual Role**: Oxygen exhibits opposite effects depending on structural context:
‐ *Global*: Ether oxygens (GenX‐type structures) increase polarizability by ∼12% per oxygen due to additional electron density and disrupted backbone conjugation (Figure S2, PC_2_ loading analysis). The 11 unstable ether‐PFAS (ΔH>0) exhibit anomalously high polarizability (229.8 ± 4.0 Bohr^3^, 99th percentile).
‐ *Local*: Oxygen atoms at sphere‐1 (carboxylate/sulfonate headgroups) decrease α‐C─F BDE by 5–12 kJ/mol through resonance stabilization of the incipient radical (Figure S3, O1 feature). This explains PFOA's lower defluorination barrier (3.1 kJ/mol) compared to PFOS (7.8 kJ/mol).
Chain Length Effects:
‐ *Global*: Longer chains (C_10__+_) exhibit higher polarizability (linear trend, +8.3 Bohr^3^/C atom) but also higher GPR uncertainty (σ^2^>150 Bohr^6^) due to undersampling in the training set. This suggests long‐chain PFAS (C_14__+_) as priority targets for active learning.
‐ *Local*: Sphere‐2 carbon counts distinguish terminal vs. internal C─F bonds: terminal bonds are 8–15 kJ/mol weaker due to reduced hyperconjugation (Figure S3, C4_sphere2 importance). This predicts that defluorination initiates preferentially at chain termini: consistent with AIMD mechanistic studies.

These cross‐property patterns enable hierarchical screening: (a) Filter by polarizability to identify high‐sorption candidates (>150 Bohr^3^) for granular activated carbon (GAC)/anion exchange removal (b) Rank by minimum BDE within the filtered set to prioritize molecules with weak C─F bonds (<110 kcal/mol) for reductive degradation (c) Flag high‐uncertainty predictions in both models for experimental validation. This integrated analysis demonstrates that even though the models operate on distinct feature spaces (3D spatial correlations vs. graph topology), they capture complementary facets of PFAS chemistry that together provide actionable remediation guidance.

### 4.3 Benchmarking Against Literature

Direct experimental benchmarks for PFAS polarizability and thermodynamic stability are scarce in the literature. Our model validation relies on internal consistency (train/test splits) and correlation with DFT‐calculated values, which themselves carry method‐dependent uncertainties (±5%–10% for B3LYP polarizabilities). For the local properties, to validate chemical accuracy, we compared predictions against AIMD‐derived barriers from Biswas et al. [22] for PFOA and PFOS. These molecules were held out from training to ensure unbiased evaluation. Table 4 shows our RF model reproduces AIMD barriers with 15% and 4% relative error, respectively. The RF model enables high‐throughput screening across thousands of molecules, infeasible with AIMD workflows. Our model's robustness was further evaluated on structurally distinct PFAS classes. Model robustness was evaluated across structurally diverse PFAS classes, including ether‐disrupted chains absent from the original Qu et al. [36] training distribution. Predictive accuracy remained consistent (MAE = 2.1 kcal/mol for ether‐linked vs. 1.8 kcal/mol for linear PFAS), confirming that topological features generalize across backbone architectures.

**TABLE 4:** Comparison of ML‐predicted Bond Dissociation Energies (BDEs) with AIMD‐calculated activation barriers from Biswas et al. [22].

Molecule	Bond Site	AIMD Barrier [kJ/mol]	Our ML‐Predicted BDE [kJ/mol]	Rel. Error
PFOA	α‐C (C7─F)	2.70 ± 0.8	3.1	∼15%
PFOS	α‐C (C7─F)	8.16 ± 1.2	7.8	∼4%
PFOS	C3─F	Not Reported	9.1	N/A
PFOS	C4─F	Not Reported	9.4	N/A

The RF model reproduces AIMD activation barriers with 15% and 4% relative error for PFOA and PFOS, respectively. Critically, the model correctly captures the chemical trend: the α‐C─F bond in PFOS is 2.5× more stable than in PFOA (7.8 vs. 3.1 kJ/mol), consistent with AIMD results (8.16 vs. 2.70 kJ/mol). This differential, predicted solely from topology, aligns with the known higher recalcitrance of sulfonates compared to carboxylates. This confirms that the framework can function as a hierarchical filter: first screening libraries for global stability, and subsequently ranking candidates by their “weakest link” (minimum BDE) to prioritize molecules most susceptible to reductive defluorination. The model also predicts BDEs across the entire fluoroalkyl chain, generating testable hypotheses (e.g., C3─F and C4─F bonds in PFOS have comparable activation barriers to α‐carbon, suggesting multi‐site defluorination pathways).

### 4.4 Implications for Remediation Strategies

The properties studied in this work translate directly into screening criteria for remediation technologies. Molecules with high polarizability (>150 Bohr^3^) and low water solubility (often correlated with hydrophobicity and chain length [20, 68]) are prime candidates for removal via adsorption on GAC or anion exchange resins [69], as both adsorption capacity and selectivity increase significantly with perfluoroalkyl chain length and molecular hydrophobicity [42, 70]. Our global property model allows for the rapid identification of these “sequesterable” candidates. Furthermore, the BDE predictions serve as a kinetic “traffic light.” C─F bonds with BDEs < ∼110–115 kcal/mol, often found near carboxylate heads or ether linkages, represent “weak points” susceptible to low‐energy defluorination methods like hydrated electron reduction [22, 71] or electrochemical reductive pathways [72, 73, 74]. Conversely, molecules where all C─F BDEs exceed 115 kcal/mol may require high‐energy destructive methods [75, 76] such as plasma treatment [77, 78], thermal incineration (>900°C) [78, 79, 80], or supercritical water oxidation. By filtering large libraries through these property thresholds, researchers can prioritize specific PFAS classes for the most appropriate treatment train.

To illustrate this hierarchical screening utility in a practical remediation scenario, consider a candidate list containing PFOA, PFOS, and a short‐chain perfluorobutane sulfonate (PFBS). In a treatment context prioritizing adsorption followed by low‐energy reductive defluorination, the workflow proceeds as follows:

1. *Filtration (Global Property)*: The model first predicts polarizability. PFOA and PFOS (longer chains) are predicted to have high polarizabilities (>150 Bohr [3]), flagging them as suitable for GAC removal, whereas PFBS (lower polarizability) is flagged for alternative sequestration methods like ion‐exchange resins.

2. *Degradation (Local Property)*: For the GAC‐amenable fraction, the model ranks candidates by minimum C─F BDE. As validated in Table 4, the model predicts that the α‐C─F bond in PFOA (3.1 kJ/mol) is significantly weaker than in PFOS (7.8 kJ/mol).

Consequently, the workflow produces a prioritized shortlist: PFOA is ranked as the prime candidate for integrated GAC‐reductive treatment, while PFOS is flagged as requiring higher‐energy input for defluorination despite similar sorption characteristics. This demonstrates how the coupled models translate molecular structure directly into process‐specific decision logic.

## 5 Conclusions

This work demonstrates that physics‐informed machine learning models can predict degradation‐relevant PFAS properties with accuracy approaching that of high‐fidelity quantum‐mechanical simulations, while enabling high‐throughput screening. By explicitly aligning feature engineering with the physical character of the target properties, 3D spatial electron distributions for global properties such as polarizability and thermodynamic stability, and local bonding topology for C─F bond dissociation energies, we obtained robust structure–property linkages across a chemically diverse PFAS dataset. The voxelization plus spatial‐correlation representation, combined with Gaussian Process Regression, achieved R^2^ = 0.92 (MAE = 9.2 Bohr^3^) for polarizability and R^2^ = 0.97 (MAE = 76.44 kJ/mol Bohr^3^) for enthalpy prediction, with well‐calibrated predictive uncertainties (empirical coverage 94.3% and 93.8% for nominal 95% CI) suitable for active learning. The graph‐based Random Forest models for local BDEs attained R^2^ = 0.87 (MAE = 1.61 kcal/mol) on PFAS semi‐empirical test sets, while preserving interpretability through chemically meaningful SHAP feature attributions.

Despite these strengths, several important limitations remain. First, the curated dataset underrepresents certain PFAS subclasses, including cyclic structures, zwitterionic species, and ultra‐long‐chain molecules (C_15_+), which leads to higher predictive uncertainty and potential extrapolation error for these chemistries. Second, the BDE labels are derived from ground‐state electronic structure calculations (PM6 and B3LYP/def2‐sv(p)) and do not directly capture solvent effects, entropic contributions, or excited‐state dynamics that govern photocatalytic pathways; consequently, the predicted BDEs should be interpreted as proxies for kinetic barriers rather than direct rate constants. Third, the current models predict properties at the molecular and bond level but do not explicitly account for process‐level variables such as reactor design, mass transport, or byproduct formation, which are essential for complete remediation workflow design. Fourth, the uncertainty quantification provided by GPR captures statistical uncertainty arising from finite and non‐uniform sampling of the input space, but does not explicitly represent systematic errors in the underlying quantum‐chemical methods (model reduction errors) or potential dataset biases.

These limitations motivate several concrete next steps. One priority is to deploy the GPR uncertainty estimates in active‐learning and multi‐fidelity frameworks that iteratively select new PFAS candidates for high‐level quantum‐mechanical calculations, focusing on regions of high predicted uncertainty or structural classes that are currently underrepresented. A second direction is to couple the local BDE predictions with transition‐state theory and Marcus‐type models of electron‐transfer kinetics, thereby translating static bond strengths into quantitative degradation rate predictions for electrochemical, radiolytic, and photocatalytic systems. A third is to carry out targeted experimental validation campaigns in collaboration with remediation practitioners, using the ML models to propose PFAS subsets and specific bond‐cleavage sites, and then using the resulting data to refine both the models and the estimated uncertainty. Finally, integrating these property predictors into inverse‐design and Bayesian optimization frameworks will enable the design of next‐generation fluorinated surfactants that retain desirable performance attributes while exhibiting intentionally weakened C─F bonds and reduced thermodynamic stability, improving their environmental degradability and reducing long‐term persistence. Longer‐term, these property predictors enable inverse design of inherently degradable fluorinated surfactants: Bayesian optimization can identify molecular structures that retain performance (e.g., surface tension, aggregation) while exhibiting intentionally weakened C─F bonds (<100 kcal/mol) and reduced thermodynamic stability (ΔH near zero). This would shift the paradigm from remediating persistent pollutants to designing transient alternatives. In summary, this work provides a validated computational infrastructure for rapid PFAS property screening that balances accuracy, efficiency, interpretability, and uncertainty quantification— essential requirements for guiding both near‐term remediation efforts and long‐term sustainable chemistry design.

## Conflicts of Interest

The authors declare no conflicts of interest.

## Supporting information




**Supporting File**: advs74195‐sup‐0001‐SuppMat.pdf.

## Data Availability

All scripts for feature engineering (voxelization and graph‐based featurization), model training (GPR and Random Forest), and post‐processing (uncertainty analysis, SHAP calculations, and figure generation) are available at https://github.com/pranoy‐ray/screenPFAS. The curated PFAS property datasets derived from the ERDC and EPA sources will be made available upon reasonable request after internal review by the data owners, subject to institutional data‐release policies.
