# Supplementary material for: ML Workflows for Screening Degradation‐Relevant Properties of Forever Chemicals
Source: Adv Sci (Weinh). 2026 Jan 31:e23817. Online ahead of print. doi: 10.1002/advs.202523817 (PMC13325644; doi:10.1002/advs.202523817)

ML workflows for screening degradation-relevant properties of forever chemicals

*Pranoy Ray[1,2], Andrew R. Castillo[3], Manoj Kolel-Veetil[4], Surya R. Kalidindi[1,2]**

[1]George W. Woodruff School of Mechanical Engineering, Georgia Institute of Technology, Atlanta, USA
[2] School of Computational Science and Engineering, Georgia Institute of Technology, Atlanta, USA
[3]Multiscale Technologies Inc., Seattle, USA
[4]United States Naval Research Laboratory, Washington DC, USA

*Corresponding author: surya.kalidindi@me.gatech.edu

Supplementary Figure S1: Graph-based Based Feature Engineering for Local Properties (Bond Dissociation Energy) employed in this work. The reference bond/atom for which the feature engineering is computed is given by the star in the molecular structure representation.

<table>
<tr><th>CAS-NO</th><th>Molecular Structure</th><th>Feature Engineering</th></tr>
<tr><td>119107-96-9</td><td>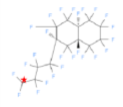</td><td>
<table>
<tr><th></th><th>C2</th><th>C3</th><th>C4</th><th>H1</th><th>N1</th><th>N2</th><th>N3</th><th>N4</th><th>O1</th><th>O2</th><th>S1</th><th>S2</th><th>S3</th><th>S4</th><th>F1</th></tr>
<tr><td>sphere0</td><td>0.0</td><td>0.0</td><td>1.0</td><td>0.0</td><td>0.0</td><td>0.0</td><td>0.0</td><td>0.0</td><td>0.0</td><td>0.0</td><td>0.0</td><td>0.0</td><td>0.0</td><td>0.0</td><td>1.0</td></tr>
<tr><td>sphere1</td><td>0.0</td><td>0.0</td><td>1.0</td><td>0.0</td><td>0.0</td><td>0.0</td><td>0.0</td><td>0.0</td><td>0.0</td><td>0.0</td><td>0.0</td><td>0.0</td><td>0.0</td><td>0.0</td><td>2.0</td></tr>
<tr><td>sphere2</td><td>0.0</td><td>0.0</td><td>1.0</td><td>0.0</td><td>0.0</td><td>0.0</td><td>0.0</td><td>0.0</td><td>0.0</td><td>0.0</td><td>0.0</td><td>0.0</td><td>0.0</td><td>0.0</td><td>2.0</td></tr>
<tr><td>sphere3</td><td>0.0</td><td>0.0</td><td>1.0</td><td>0.0</td><td>0.0</td><td>0.0</td><td>0.0</td><td>0.0</td><td>0.0</td><td>0.0</td><td>0.0</td><td>0.0</td><td>0.0</td><td>0.0</td><td>2.0</td></tr>
</table>
</td></tr>
<tr><td>118334-94-4</td><td>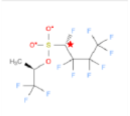</td><td>
<table>
<tr><th></th><th>C2</th><th>C3</th><th>C4</th><th>H1</th><th>N1</th><th>N2</th><th>N3</th><th>N4</th><th>O1</th><th>O2</th><th>S1</th><th>S2</th><th>S3</th><th>S4</th><th>F1</th></tr>
<tr><td>sphere0</td><td>0.0</td><td>0.0</td><td>1.0</td><td>0.0</td><td>0.0</td><td>0.0</td><td>0.0</td><td>0.0</td><td>0.0</td><td>0.0</td><td>0.0</td><td>0.0</td><td>0.0</td><td>0.0</td><td>1.0</td></tr>
<tr><td>sphere1</td><td>0.0</td><td>0.0</td><td>1.0</td><td>0.0</td><td>0.0</td><td>0.0</td><td>0.0</td><td>0.0</td><td>0.0</td><td>0.0</td><td>0.0</td><td>0.0</td><td>0.0</td><td>1.0</td><td>1.0</td></tr>
<tr><td>sphere2</td><td>0.0</td><td>0.0</td><td>1.0</td><td>0.0</td><td>0.0</td><td>0.0</td><td>0.0</td><td>0.0</td><td>2.0</td><td>1.0</td><td>0.0</td><td>0.0</td><td>0.0</td><td>0.0</td><td>2.0</td></tr>
<tr><td>sphere3</td><td>0.0</td><td>0.0</td><td>2.0</td><td>0.0</td><td>0.0</td><td>0.0</td><td>0.0</td><td>0.0</td><td>0.0</td><td>0.0</td><td>0.0</td><td>0.0</td><td>0.0</td><td>0.0</td><td>2.0</td></tr>
</table>
</td></tr>
<tr><td>105923-79-3</td><td>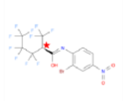</td><td>
<table>
<tr><th></th><th>C2</th><th>C3</th><th>C4</th><th>H1</th><th>N1</th><th>N2</th><th>N3</th><th>N4</th><th>O1</th><th>O2</th><th>S1</th><th>S2</th><th>S3</th><th>S4</th><th>F1</th></tr>
<tr><td>sphere0</td><td>0.0</td><td>0.0</td><td>1.0</td><td>0.0</td><td>0.0</td><td>0.0</td><td>0.0</td><td>0.0</td><td>0.0</td><td>0.0</td><td>0.0</td><td>0.0</td><td>0.0</td><td>0.0</td><td>1.0</td></tr>
<tr><td>sphere1</td><td>0.0</td><td>1.0</td><td>2.0</td><td>0.0</td><td>0.0</td><td>0.0</td><td>0.0</td><td>0.0</td><td>0.0</td><td>0.0</td><td>0.0</td><td>0.0</td><td>0.0</td><td>0.0</td><td>0.0</td></tr>
<tr><td>sphere2</td><td>0.0</td><td>0.0</td><td>1.0</td><td>0.0</td><td>0.0</td><td>1.0</td><td>0.0</td><td>0.0</td><td>0.0</td><td>1.0</td><td>0.0</td><td>0.0</td><td>0.0</td><td>0.0</td><td>5.0</td></tr>
<tr><td>sphere3</td><td>0.0</td><td>1.0</td><td>1.0</td><td>1.0</td><td>0.0</td><td>0.0</td><td>0.0</td><td>0.0</td><td>0.0</td><td>0.0</td><td>0.0</td><td>0.0</td><td>0.0</td><td>0.0</td><td>2.0</td></tr>
</table>
</td></tr>
</table>

Supplementary Figure S2: Analysis for prediction of global properties in this work: (a), (b) and (c) are the MAE v/s chain architecture, MAE v/s Chain backbones and Cross-validation training plots respectively, for the Sum of Free Energies and Enthalpies. (d), (e) and (f) are the same evaluations for Polarizability.

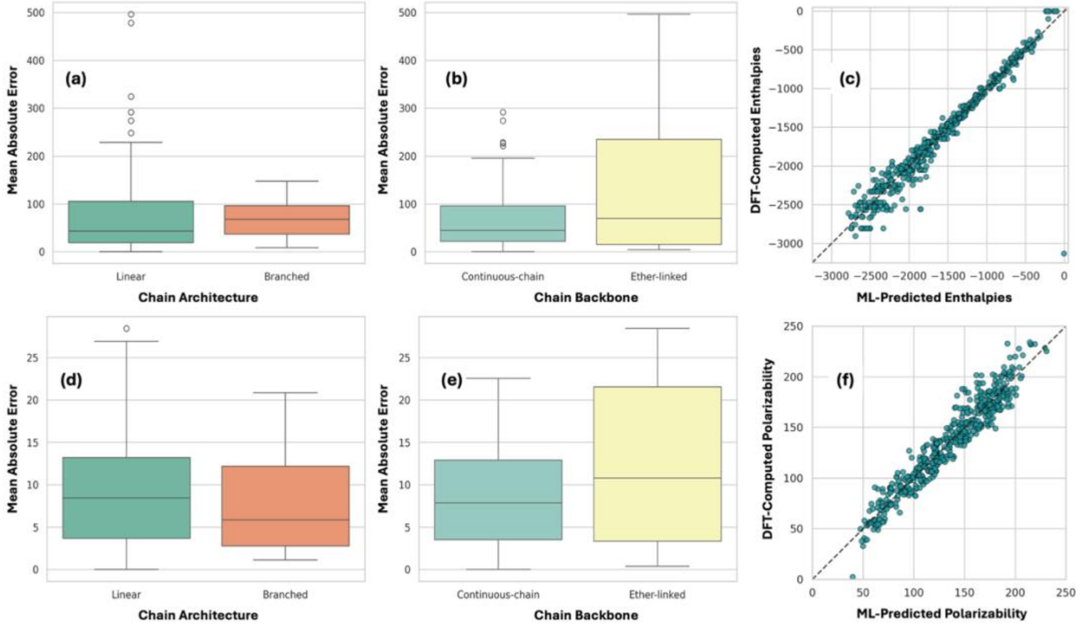

Supplementary Figure S3: SHAP analysis for prediction of Bond Dissociation Energy employed in this work.

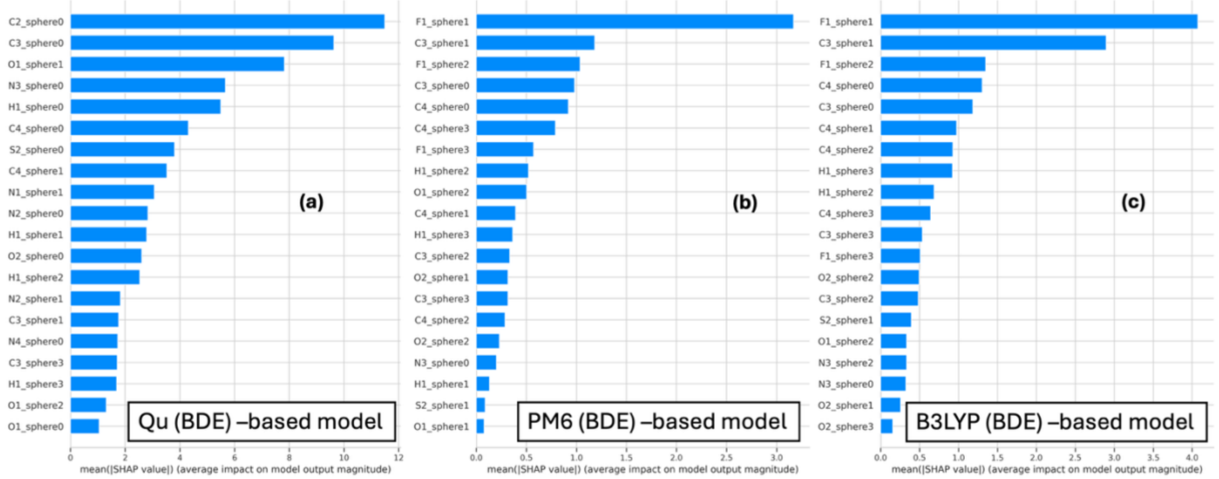

Supplement: Supplementary file 1 — Supporting File: advs74195‐sup‐0001‐SuppMat.pdf. [file ADVS-9999-e23817-s001.pdf]
